# Growth phase proteomics of the heterotrophic marine bacterium *Ruegeria pomeroyi*

**DOI:** 10.1038/s41597-019-0308-y

**Published:** 2019-12-03

**Authors:** Dasha Krayushkina, Emma Timmins-Schiffman, Jessica Faux, Damon H. May, Michael Riffle, H. Rodger Harvey, Brook L. Nunn

**Affiliations:** 1 University of Washington, Department of Psychiatry and Behavioral Sciences, Seattle, WA 98195 USA; 2 University of Washington, Department of Genome Sciences, Seattle, WA 98195 USA; 3 Old Dominion University, Department of Ocean, Earth & Atmospheric Sciences, Norfolk, VA 23529 USA; 4 University of Washington, Department of Biochemistry, Seattle, WA 98195 USA

**Keywords:** Proteomics, Bacterial physiology, Proteomic analysis

## Abstract

The heterotrophic marine bacterium, *Ruegeria pomeroyi*, was experimentally cultured under environmentally realistic carbon conditions and with a tracer-level addition of ^13^C-labeled leucine to track bacterial protein biosynthesis through growth phases. A combination of methods allowed observation of real-time bacterial protein production to understand metabolic priorities through the different growth phases. Over 2000 proteins were identified in each experimental culture from exponential and stationary growth phases. Within two hours of the ^13^C-labeled leucine addition, *R*. *pomeroyi* significantly assimilated the newly encountered substrate into new proteins. This dataset provides a fundamental baseline for understanding growth phase differences in molecular physiology of a cosmopolitan marine bacterium.

## Abstract (summary)

**Table:** 

Measurement(s)	proteomic profiling
Technology Type(s)	liquid chromatography-tandem mass spectrometry
Factor Type(s)	sample interval • biological replicate
Sample Characteristic - Organism	Ruegeria pomeroyi
Sample Characteristic - Environment	marine biome

Machine-accessible metadata file describing the reported data: 10.6084/m9.figshare.10282922

## Background & Summary

Ecosystems are sustained by biogeochemical cycling of major elements essential to organism survival. Principally driven by microbes, elemental cycling pathways depend on processes including the uptake and incorporation of inorganic nutrients, consumption by heterotrophic consumers, and degradation and remineralization of organic material. Proteins catalyze and mediate these processes essential for organism-environment interaction, making them windows into ecosystem health and function^1^.

Protein synthesis and consumption essential to many marine biogeochemical processes are difficult to fully explore due to the limited ability to examine proteins *in situ*. To minimize this limitation, the rapidly advancing field of proteomics is providing insights into many processes that have not previously been examined^2–4^. Bacterial proteins are often masked by the more abundant inputs of eukaryotic marine organisms, making it technically difficult to detect the full suite of active bacterial processes in mixed environmental samples^5–10^. Datasets that provide a foundation to better examine ecosystem cycling, degradation, and preservation of proteins that emphasize bacteria’s role in these processes are necessary to improve current understanding of biogeochemical cycling. Understanding diverse bacterial functional roles in the environment will require surveys of natural communities coupled with detailed, in-depth analyses of cultured species. Through high resolution mass spectrometry-based proteomics, we present the proteome of *Ruegeria pomeryoi* through cell cycle phases in realistically low carbon oceanic conditions. Further, we expand the environmentalist’s molecular toolbox of methods by demonstrating how proteomic profiling of *in situ* ^13^C incorporation into newly synthesized proteins can inform us about the timing of different cellular metabolic processes.

*Ruegeria pomeroyi* is a marine heterotrophic bacterium that, in part due to its generalist nature, has become the foundation of many lab manipulations to understand bacteria-environment interactions^11,12^. *R*. *pomeroyi’s* sequenced genome makes it an ideal species for detailed investigations, such as proteomics. Fifty percent of the *R*. *pomeroyi* genome is considered sensitive to changing transcript abundance in response to abiotic environmental change^11^. Although proteomic profiles and cellular physiology are known to shift throughout bacterial culture experiments due to depletion in local nutrients, space, and oxygen^13,14^, the underlying molecular physiological response is poorly understood through the progression of different growth phases.

**Fig. 1:**
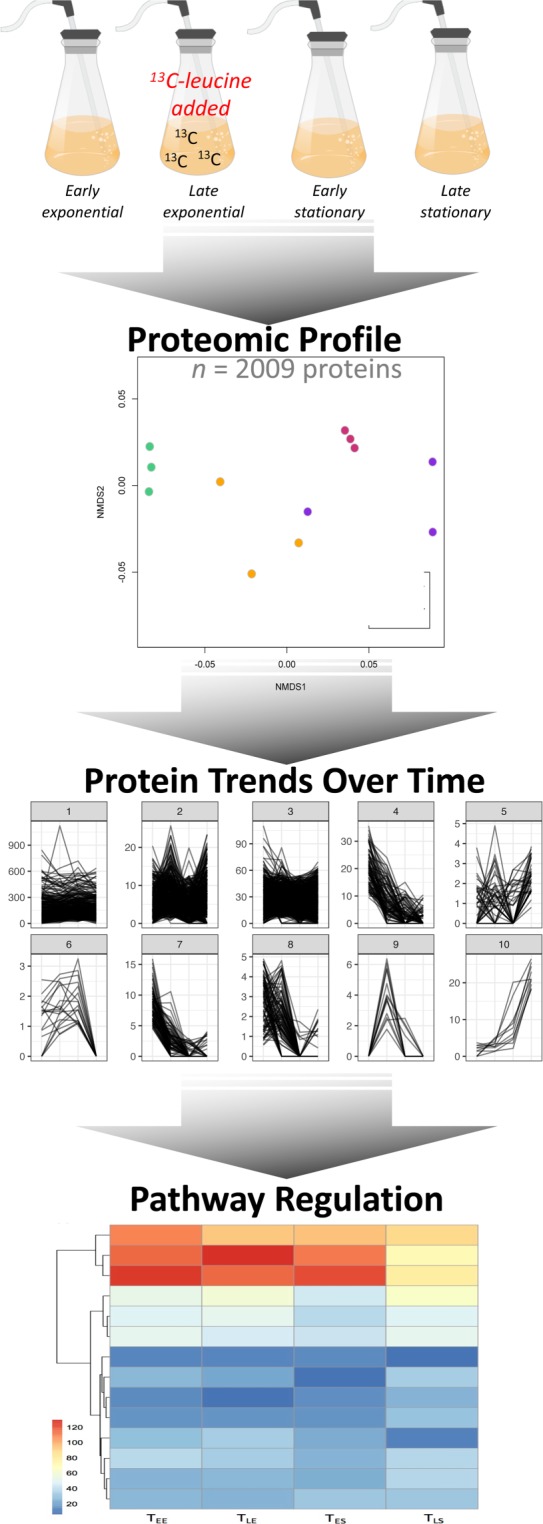
Schematic of experimental design and applications for data use. Proteomes were sequenced from four growth phases of *R*. *pomeroyi*. These data can later be used to discover trends of protein abundance over time and how different molecular pathways are regulated throughout bacterial growth.

It is essential to understand molecular level metabolic alterations to controlled, realistic growth conditions through the growth phases if we are to truly characterize how species respond to *in situ* environments and perturbations. Here, we thoroughly characterize the proteome of *Ruegeria pomeroyi*, a member of the Roseobacter clade, using a mass spectrometry technology capable of sequencing relatively low abundance proteins to better understand the baseline physiology of a heterotrophic marine bacterium across growth phases (Table 1). We employed whole proteome profiling, or data dependent acquisition proteomics, to uncover complete pathways and how they contribute to different growth phases (Fig. 1). In addition, *R*. *pomeroyi in situ* protein synthesis was tracked using trace amounts of ^13^C-labeled leucine.

**Table 1:** Description of samples used in analysis. “DDA proteomics” refers to data dependent acquisition proteomics, or whole proteome profiling.

Growth Phase	Protocol 1	Protocol 2	Dataset Identifier
Early Exponential	DDA proteomics		PXD008661^27^
Late Exponential	DDA proteomics	^13^C-leucine	PXD008661^27^
Early Stationary	DDA proteomics		PXD008661^27^
Late Stationary	DDA proteomics		PXD008661^27^

## Methods

### Bacterial culture and growth

*R*. *pomeroyi* DSS-3 was maintained on a marine basal media (28 salinity) with vitamin supplements and 10 mM glucose as the sole carbon source. Cultures were kept in continual culture, transferring every 48–72 hours once glucose concentrations were depleted to <0.1 mM.

Experimental cultures were sampled at 8-hour intervals for 48 hours to determine growth dynamics. Each experimental growth curve was established for *R*. *pomeroyi* by spectrophotometric analysis at an optical density of 600 nm (OD_600_) and calibrated using 5 mL sub-samples preserved in 2% formalin and filtered onto black 0.2 µm polycarbonate filters for cell counts. Fluoroshield with DAPI histology mounting medium (Sigma-Aldrich, St. Louis, MO) was used to stain cells for direct counting^15^. Cells were visualized and counted with an Olympus BX50 microscope (Shinjuku, Tokyo) at 1250x magnification.

For proteomics sampling across growth phases, three replicate experimental cultures in 2 L flasks (and a 1 L media blank) were grown in marine basal media with 0.1 mM glucose. Three separate 2 L replicates were also grown using stable isotope probing to track assimilation of a dissolved free amino acid into new proteins at early exponential and late exponential growth phases. ^13^C_6_-labeled leucine (^13^C-Leu, Sigma-Aldrich) substrate was added 23 hours after inoculation, (616 nM) and the late exponential culture harvested 2 hours later to capture protein synthesis during exponential growth. Based on measured cellular growth rates and the high and low estimates for carbon content per cell in marine bacteria^16^, this avoided ^13^C-Leu depletion prior to the next sample time point (36 hrs) (Figshare File 1^17^). To capture protein synthesis during late exponential growth, a second addition of ^13^C-Leu was added at 36 hours and the culture harvested 2 hours later to track ^13^C-labeled proteins that remained plus newly synthesized ^13^C-proteins.

### Sampling procedures for proteomics

Triplicate bacterial cultures were sampled for proteins at 25 hours, 38 hours, 44 hours, and 66 hours, corresponding to early exponential, late exponential, early stationary, and late stationary growth phases, respectively. To track *in situ* newly synthesized proteins, triplicate cultures with the ^13^C-leucine label addition were sampled at 25 hours and 38 hours. Samples were taken from each culture flask for data dependent acquisition (DDA) proteomic analysis with the volumes adjusted to collect similar protein amounts over cellular growth. A 10% (w/v; 4 °C) solution of trichloroacetic acid (TCA) was added to each sample to rapidly kill the bacteria and precipitate the proteins. Samples were refrigerated at 4 °C for 1 hour prior to filtration through a 0.2 µm polycarbonate filter and frozen at −80 °C until extraction.

### Protein digestion

Cells were isolated by placing the filters in 1.5 mL tubes with 300 µL of 2 M urea. Samples were placed on ice and shaken on a bead beater 5 times (1 minute each) to shake the cells off the filters. The filters were removed and the cells were lysed using a microtip sonicator (Branson, Danbury, CT) in 5 rounds, 15 seconds for each sonication, placing on ice between sonication events.

Samples were digested following [18]. Briefly, proteins were reduced with 2.5 µl of 200 mM tris(2-carboxyethyl)phosphine for 1 hour at 37 °C. Reduced proteins were then alkylated with 20 µl of 200 mM iodoacetamide for 1 hour in the dark. Ammonium bicarbonate was added to dilute the urea, followed by an addition of methanol to increase the solubility of membranes. Trypsin was administered to samples at an enzyme-to-protein ratio of 1:50. Samples were vortexed and incubated overnight at 37 °C. Liquid was evaporated in a speed vac and dried peptides were reconstituted in 5% acentonitrile (ACN) + 0.1% trifluoroacetic acid.

A macro-spin C18 column (NestGroup, Southborough, MA) was used to desalt digested peptide samples following the manufacturer’s instructions. Desalted peptides were dried via speedvac and 0.5 µg was brought up to a standard volume with 5% ACN with 0.1% formic acid (FA) for analysis by tandem mass spectrometry (MS/MS).

### LC-MS/MS: Liquid chromatography and tandem mass spectrometry

Tandem mass spectrometry (LC-MS/MS) was performed on a Q-Exactive (QE) mass spectrometer (Thermo Fisher). Peptides were chromatographically separated on a 20 cm long, 75 µm id fused silica capillary column packed with C18 particles (Magic C18AQ, 100 Å, 5 µm; Bruker, Billerica, MA) preceded by a pre-column (2 cm length, 100 µm; Magic C18AQ, 200 Å, 5 µm, Bruker). Peptides were eluted with an acidified (FA, 0.1% w/v) water-ACN gradient (5–35% ACN) over 90 minutes using a nanoAcquity Ultra Performance LC (Waters; Milford, MA) at 0.25–0.3 µl/min in line with the QE. QE settings included a 30.0 s dynamic exclusion, full MS resolution of 35000, full MS AGC target of 5e5, full MS maximum IT of 100 ms, full MS scan range of 400–1600 *m/z*, MS/MS resolution of 17500, MS/MS AGC target of 5e4, MS/MS maximum IT of 50 ms, MS/MS loop count of 20, MS/MS MSX count of 1, MS/MS isolation window of 2 *m/z*, and MS/MS NCE of 25.0.

Raw files were searched against the *R*. *pomeroyi* proteome (http://www.uniprot.org/proteomes/UP000001023, downloaded July 11, 2013) with added common laboratory contaminants (http://www.thegpm.org/crap/) using Comet v. 2016.01 rev. 2^18,19^. The final protein database contained 4260 predicted *R*. *pomeroyi* protein sequences and 4531 sequences total. All Comet settings can be found in the comet.params file (Figshare File 2^17^) and included a concatenated decoy search, peptide mass tolerance of 2.1 amu, fully trypsin digested peptides, 2 allowed missed cleavages, variable modification of 15.9949 on methionine with a maximum of 5 per peptide, fragment bin tolerance of 1.0005 and fragment bin offset of 0.4. The Trans-Proteomic Pipeline (Peptide and ProteinProphet) was run on all files with a probability cut-off of 0.9 (corresponding to a false discovery rate of about 0.0035) for peptide and protein inferences^20,21^. Abacus^22^ was used to calculate normalized spectral abundance factor (NSAF) values and find consensus protein inferences across all pep.xml and prot.xml files (Figshare File 3^17^). Stringent cut-offs were chosen for Abacus parameters to ensure inclusion of high confidence proteins in the dataset. Parameters for Abacus included 0.99 probability for the minimum PeptideProphet score of a best peptide match for a protein; 0.50 probability for the minimum PeptideProphet score for a peptide to be considered by Abacus; 0.90 probability for a protein group ProteinProphet score. The Abacus output for proteins with at least 2 unique spectral counts across replicates was used for downstream analyses (Figshare File 4^17^). The resulting dataset contained 2009 proteins, or 47% of the predicted *R*. *pomeroyi* proteome, which is within the range of proteome coverage for other bacterial proteomes^23–25^. All proteomics data are available via the ProteomeXchange Consortium via the PRIDE partner repository^26^, accession number PXD008661^27^.

^13^C-leucine samples were similarly searched, except the following setting was changed in the comet.params file: variable_mod02 = 6.0 L 0 3–1 0 0, allowing the searching algorithm to identify peptides with leucine with or without the modification of +6, indicative of the 6- ^13^carbons that were included in the leucine tracer experiment. TPP and Abacus were deployed as described above (Figshare File 5^7^).

### Label-free protein quantification and statistical analysis

Peptide spectral counts per protein were used to estimate relative abundance of proteins for each sample in the unlabeled experiment. This was performed under the accepted assumption that the more abundant a protein is within a sample, the more peptides it will generate upon digestion, thus allowing for more spectra to be detected from those peptides^28^. Contaminant proteins were removed from the data prior to analysis.

A nonmetric multidimensional scaling (NMDS) analysis was performed on the NSAF data for the growth curve experiment using a Bray-Curtis dissimilarity matrix with log(x + 1)-transformed data in the vegan package^29^ in R (https://www.R-project.org/). NMDS was used to quantify and visualize the pairwise dissimilarity in protein composition between samples of each time point.

## Data Records

Figshare File 1^17^: Calculations used to derive the amount of ^13^C-labeled leucine incorporated into cells. File is in .docx format.

Figshare File 4^17^: Proteomics data from the growth experiment (the proteomics without ^13^C label). The first column contains the protein identifier (Uniprot accession), followed by the protein length, then the total number of unique peptides detected for that protein across all biological and technical replicates (‘No. Unique Peptides Across All Replicates). The subsequent columns contain the total spectral counts and Normalized Spectral Abundance Factor values for each mass spectrometry analysis, (e.g., Early Exp 1, the first technical replicate for early exponential stage). Table is in .xlsx format.

Figshare File 5^17^: Proteomics data from the ^13^C-labeled leucine experiment. The data are arranged similarly to Figshare File 4, except the last 4 columns are indicators of label incorporation into the protein. An asterisk in the column indicates that a peptide with a ^13^C label was detected for that protein. Table is in .xlsx format.

Figshare File 6^17^: Raw proteomics mass spectrometry files record. Files were deposited in the online repository PRIDE under the ProteomExchange with identifier PXD008661^27^. The first column of the table contains the file name, the second column is the file format, third is how the file was produced, and fourth is the growth stage the data are derived from. Table is in .xlsx format.

## Technical Validation

**Fig. 2:**
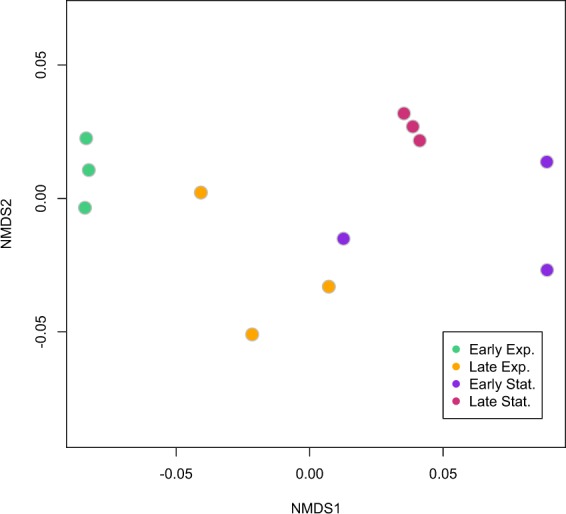
Nonmetric multidimensional scaling plot of the growth experiment (*R*. *pomeroyi* incubated without ^13^C) proteomics data. Each point represents the NSAF values across the entire proteome for a sample and points fall closer together if they have more similar proteomic profiles. Color indicates growth phase: Early Exponential = green, Late Exponential = orange, Early Stationary = purple, Late Stationary = pink.

Technical and biological replication (reproducibility on the mass spectrometer and within time points) were assessed using NMDS (Fig. 2). There was generally clustering among time point replicates, with the exception of one of the early stationary phase points (purple) that clustered closer to late exponential phase samples than other early stationary phase samples. Throughout the mass spectrometry experiment, quality control standards were run between sets of samples to ensure reproducibility of the mass spectrometry data.

## Data Availability

Figshare File 2^17^: Parameters file used for Comet searches of mass spectrometry data. File is in .docx format. Figshare File 3^17^: Parameters file used for Abacus analysis of Comet search results. File is in .docx format.
